# What Do We Know About Proximal Factors Influencing Bystander Intervention? A Call for Momentary Assessment and Intervention

**DOI:** 10.1007/s10935-026-00903-z

**Published:** 2026-03-17

**Authors:** Daniel W. Oesterle, Molly Maloney, Kennicia Fortson, Annelise Mennicke, Erika Montanaro

**Affiliations:** 1 Department of Psychological Sciences, Purdue University, 703 Third Street, West Lafayette, IN 47907 USA; 2 Alpert Medical School of Brown University, Providence, RI USA; 3 School of Public Health, Brown University, Providence, RI 02903 USA; 4 Violence Prevention Center, College of Health & Human Services, University of North Carolina Charlotte, Charlotte, NC USA; 5 School of Social Work, College of Human & Health Services, University of North Carolina Charlotte, Charlotte, NC USA; 6 Department of Psychological Science, University of North Carolina Charlotte, Charlotte, NC USA

**Keywords:** Bystander intervention, Prosocial behavior, Methods, Alcohol, Prevention, Sexual violence

## Abstract

Bystander intervention (BI) is among the most extensively studied phenomena in social psychology, with theoretical models underscoring a range of situational factors that influence helping behavior. Yet, despite considerable attention and emphasis placed on understanding these processes, we know very little about momentary and dynamic factors that effect actual BI in real-time. This is especially problematic because BI prevention programs are designed to promote in-the-moment behavioral responses to emergent risk, yet the evidence base supporting their use relies predominantly on cross-sectional retrospective self-reports, intervention-likelihood paradigms, and other proxy outcomes with limited external and ecological validity. Due to longstanding methodological constraints, our collective knowledge of how proximal and situational factors directly influence real-world BI behavior remains limited, yet understanding how event-level influences such as acute alcohol intoxication impact BI is essential for creating effective sexual violence prevention programming. In this debate paper, we synthesize the current literature on proximal influences of BI and propose a framework to guide future research using ecologically valid, real-time momentary assessment methods.

Bystander intervention (BI) is a common approach to promoting prosocial behavior and represents one of the most widely-examined social-psychological phenomena in the past century. Rigorous evaluation efforts and meta-analytic findings provide compelling evidence that BI is an efficacious and effective strategy for preventing sexual violence by increasing awareness of contextual risk cues, enhancing motivation for proactive intervention, and providing opportunity for skill acquisition and practice (e.g., Coker et al., 2016; Jouriles et al., 2018; Kettrey & Marx, 2019; Park & Kim, 2023); however, the scope of this evidence remains limited, as most evaluations focus on creating shifts in bystander attitudes, intentions, confidence, and self-efficacy following program participation (Bush et al., 2019). Similarly, existing BI prevention programs have been criticized as primarily impacting *intentions* to intervene, as opposed to influencing rates of BI action or reducing violence within communities (Porat et al., 2024). Given the well-documented intention–behavior gap in behavior-change research (Sheeran & Webb, 2016), a focus on bystander attitudes and intentions provides limited insight into whether—and under what conditions—intervention actually occurs. Seminal findings from Darley & Latané’s (1968) research emphasized numerous individual-difference and situational barriers to intervening, including diffusion of responsibility, evaluation apprehension, and pluralistic ignorance. More recent research has identified a plethora of other barriers and facilitators to BI (Bennett & Banyard, 2014; Oesterle et al., 2018; Orchowski et al., 2024); however these findings are limited in their real-world applicability. Given the complex, dynamic, and multifaceted nature of sexual violence, effective prevention efforts require empirical research that elucidates the real-world, proximal mechanisms and contextual factors shaping actual BI behavior; ecological momentary assessment (EMA) is uniquely positioned to address this need by capturing real-time data within the high-risk contexts in which BI decisions occur.

## Existing Research on Proximal Factors Influencing BI

Research has begun to move beyond descriptive cataloguing of barriers and facilitators to BI (e.g., Park et al., 2024) to more advanced experimental approaches that account for time-varying effects (e.g., Clay-Warner et al., 2024; Huang et al., 2023). These studies demonstrate that changing contextual and situational factors (e.g., gender, severity, etc.) influence likelihood intentions of using BI. Yet even these experimental approaches are unable to successfully replicate the complexities of real-life situations, making it difficult to determine the *real-world* proximal determinants of BI. Although experimental analogues can assess the temporal dynamics of situational processes and BI in a controlled setting, they fundamentally fail to replicate the acute stress, anxiety, and fear inherent in real-world emergencies, often rendering them ineffective at eliciting authentic levels of affective and emotional intensity (Gray et al., 2017). Even when examining real-time substance use, existing studies have operationalized BI using outcomes that remain psychologically proximal but behaviorally distal, rather than capturing intervention as it occurs in-vivo (Leone et al., 2025; Wiseblatt et al., 2025).

## Role of Proximal Risk Factors in Theoretical Models

The Situational Model (Burn, 2009)—which is one of the most widely used explanatory frameworks for BI—outlines a sequence of decisions that a bystander must make before acting to prevent or stop sexual violence: (a) noticing the event, (b) interpreting it as problematic, (c) assuming responsibility, (d) knowing how to intervene, and (e) taking action. While existing prevention programs often draw on this model to address barriers to intervention, critics have noted its limited applicability to more complex scenarios involving friends or acquaintances, wherein social dynamics and relational factors may inhibit intervention (Banyard, 2015). In response, alternative theoretical models—such as the Theory of Action Coils (Banyard, 2015) and the Bystander Intervention for Problematic Alcohol Use Model (Mennicke et al., 2024, 2025)—have emphasized the convergence of decision-making processes, situational complexity, and event-level characteristics in promoting bystander behavior. Although these innovative frameworks offer meaningful theoretical and conceptual contributions to our understanding of BI, theoretical models that explicitly incorporate acute alcohol intoxication as a momentary event-level barrier—despite its frequent co-occurrence within high-risk contexts for sexual violence (Abbey et al., 2004; Cleveland et al., 2019)—remain underdeveloped. Metatheoretical frameworks such as the I³ Model (Finkel & Hall, 2018), however, may provide a useful organizational template for researchers to examine the interactive effects of affective, behavioral, and cognitive processes on BI under varying levels of intoxication (Finkel & Eckhardt, 2013; Parrott & Eckhardt, 2018). Therefore, advancing BI theory and prevention requires identifying for *whom*, and *under what momentary conditions*, proximal cognitive, affective, and contextual factors—including acute alcohol intoxication—interact to facilitate or inhibit real-world bystander behavior, requiring the use of innovative methods to capture dynamic these processes as they unfold.

## Ecological Momentary Assessment for BI

Ecological momentary assessment offers a promising framework for studying bystander behavior as it unfolds in everyday life. EMA is an innovative, real-world, momentary assessment method developed to observe state-level constructs—such as behavioral, experiential, and affective processes—within ecologically-valid contexts (Shiffman et al., 2008; Stone & Shiffman, 1994). As opposed to retrospective self-report methods, EMA relies on delivering brief assessments over repeated narrow time intervals, intended to decrease bias and error that result from memory deficits and other cognitive limitations (Stone et al., 2023).

Researchers have successfully used EMA to study alcohol use—a key proximal factor influencing BI—among varying populations (Dvorak et al., 2014; Remmerswaal et al., 2019), exploring precipitants and motives for drinking and assess situational-level predictors of alcohol-related consequences (Wray et al., 2014), including sexual risk behaviors and sexual violence (Grocott et al., 2024; Yeater et al., 2022). Although prior research has examined the relationship between acute intoxication and BI qualitatively (Oesterle et al., 2018; Temple et al., 2024), retrospectively (Orchowski et al., 2016), and experimentally (Leone & Parrott, 2019), no research has yet explored how alcohol and substance use—alongside other event-level factors—proximally predicts BI behaviors in ecologically-valid contexts using ambulatory assessment methods.

Findings from studies using EMA also lay the foundation for developing and implementing innovative and ecologically valid prevention interventions, such as ecological momentary interventions (EMIs). EMIs are interventions designed to provide real-time support to individuals in their daily and naturalistic contexts, often using smartphones (Heron & Smyth, 2010). EMIs have demonstrated promise in reducing alcohol use (Suffoletto et al., 2012; Wright et al., 2018) and represent an innovative way to deliver BI programming. EMAs can be integrated with EMIs or Just-In-Time Adaptive Interventions (JITAIs) to target real-time barriers and facilitators to BI, offering an ideal approach to bridging the intention-behavior gap (Sheeran & Webb, 2016) by delivering interventions precisely *when* and *where* opportunities arise (Nahum-Shani et al., 2018). EMIs or JITAIs are also able to address the effects of alcohol use on social information processing and behaviors in ways not previously possible. For example, EMIs can disrupt the acute impacts of alcohol intoxication by identifying and encouraging BI (Bennett et al., 2014). Because they are often delivered via smartphone, 91% of which adults in the US own (Pew Research Center, 2024), and are responsive to individuals’ needs and contexts, EMIs are also highly scalable and avoid lack of engagement characteristic of other digital interventions (Blayney et al., 2018). Accordingly, the next generation of BI research should integrate EMA with EMIs and JITAIs—allowing researchers to move beyond simply documenting and cataloging risk toward actively disrupting risk pathways—by identifying *when*, for *whom*, and through *which* momentary intervention strategies bystander intentions can be converted into real-world prosocial behavior.

## Limitations in Using EMA for BI

This article is intended as a conceptual “*call to arms*”, urging preventionists and researchers using BI approaches to move beyond static, retrospective designs, and rather toward the use of proximal, momentary approaches capable of capturing the dynamic conditions under which prosocial behavior unfolds. As a conceptual and narrative review rather than a systematic synthesis, the present paper does not aim to exhaustively capture all available studies and may be subject to selection bias in the literature highlighted; accordingly, several limitations related to both the scope of the review and the use of momentary approaches to study BI in ecologically-valid contexts warrant further discussion. Proximal determinants of BI—including affect, intoxication, peer context, and perceived social pressure—are highly dynamic and infrequent, posing challenges for efficient sampling, construct validity, and participant burden. Moreover, the integration of EMA with EMIs or JITAIs remains largely theoretical in BI contexts, and additional methodological work is needed to establish best practices for sampling intervals, compliance thresholds, and analytic strategies. EMA research conducted in high-risk or substance-involved contexts also raises ethical concerns related to data reliability, equity, confidentiality and data security, as well as ambiguity surrounding researcher responsibility during periods of acute risk or intoxication and the complexity of obtaining informed consent in dynamic, real-world settings. Addressing these limitations is essential to ensure that EMA-based BI research is scientifically rigorous, ethically sound, and capable of advancing real-world prevention.

## Practical, Translational, & Implementation-Related Considerations

To address these challenges, researchers studying BI through EMA may increase the feasibility, acceptability, and implementation effectiveness by using a progressively-staged, context-sensitive, implementation strategy. Before deploying intensive event-level designs, researchers may use mobile micro-surveys administered around high-risk social contexts (e.g., campus parties, nightlife venues, athletic events) to first identify and validate putative proximal determinants of BI within ecologically-relevant settings. In fact, key correlates known to fluctuate in these contexts—including affect (Wray et al., 2014), peer composition (Yeater et al., 2022), intoxication (Leone et al., 2025), opportunity recognition (Orchowski et al., 2024), and perceived safety or social pressure (Temple et al., 2024)—all can be assessed using low frequency, cross-sectional surveys to establish initial construct validity prior to more intensive momentary sampling. Adaptive sampling strategies—including random-time, event-contingent, and location-based prompts (e.g., geofencing and Bluetooth-triggered sensing)—can target periods of heightened risk while minimizing irrelevant assessments to reduce participant burden and increase engagement (Heron & Smyth, 2010; Nahum-Shani et al., 2018; Rabbi et al., 2015). While not yet tested in BI contexts, existing prevention research suggests that JITAIs delivered via smartphone notifications, passive sensing, and personalized reinforcement schedules can enhance engagement and deliver support precisely when barriers or opportunities for intervention arise (Suffoletto et al., 2012; Wright et al., 2018). Given the sensitive and potentially high-risk nature of studying BI in the context of preventing sexual violence, ethical and safety considerations must be embedded throughout, including clear opt-out options, encrypted data collection, and automated access to support and safety-related resources during distress or moments of imminent risk (Hoelscher et al., 2025). Collectively, these strategies may address many of the aforementioned limitations and ethical concerns, and support scalable, prevention infrastructures capable of monitoring dynamic risk and promoting real-world bystander action.

## Public Health & Institutional Prevention Implications

In addition to practical considerations related to intervention implications, future research addressing proximal and event-level processes may also have profound implications for shaping public health and institutional approaches to sexual violence prevention. In fact, collecting and understanding how momentary data is related to specific situational patterns—such as *where*, *when*, and *under what conditions* bystanders are most or least likely to intervene—can directly inform targeted prevention policies used within high-risk settings (e.g., campus events, nightlife venues, athletic environments, or military installations). Further, these data can provide insight into intervention timing (e.g., messaging before weekends or social events), previously unidentified risk contexts, allocation of viable prevention resources (e.g., sober monitors in high-risk contexts), and policy revisions (e.g., revising alcohol service protocols, event safety procedures, or mandatory reporting policies). At a broader level, this momentary approach supports a shift in prevention towards idiographic and precision interventions—individually-informed interventions that dynamically adapt to context, environmental risk, and social dynamics—aligning sexual violence prevention with adjacent ongoing public health priorities.

## Conclusion

BI is a widely-used sexual violence prevention strategy; however, little is known about the momentary and event-level effects of proximal barriers and facilitators for BI. EMA is a particularly promising approach for studying event-level dynamics surrounding BI, allowing for a fine-grained analysis of how barriers and facilitators change over time and within contexts, which may inform innovative momentary prevention opportunities. Ultimately, adopting more ecologically valid, real-time assessment and intervention strategies has the potential to shift the field from merely enhancing BI *intentions* to driving the actual enactment of BI *behaviors* that prevent violence.

## Data Availability

There is no data to share as the current manuscript provides a review of existing published studies.
